# Improvement of Cardiometabolic Control with Dapagliflozin in Patients with Type 2 Diabetes in Primary Care: The AGORA-AP Study

**DOI:** 10.3390/medicina61122087

**Published:** 2025-11-23

**Authors:** Vicente Pallarés-Carratalá, Antonio Ruiz-García, Antonio Segura Fragoso, Carlos Escobar-Cervantes, María Inmaculada Cervera-Pérez, Francisco Javier Alonso-Moreno, Ezequiel Arranz-Martínez, Alfonso Barquilla-García, Daniel Rey-Aldana, José Polo-García, Sergio Cinza-Sanjurjo

**Affiliations:** 1 Department of Medicine, Jaume I University, 12071 Castellon, Spain; 2 Centro de Salud Pinto, 28320 Madrid, Spain; antoniodoctor@gmail.com; 3 Epidemiology Unit, Semergen Research Agency, 28009 Madrid, Spain; asegurafr@gmail.com; 4 Cardiology Department, University Hospital La Paz, 28046 Madrid, Spain; carlos.escobar@salud.madrid.org; 5 Consultorio Auxiliar Tendetes, 46009 Valencia, Spain; inmacerveraperez@gmail.com; 6 Centro Salud Sillería, 45001 Toledo, Spain; falonsom@semergen.es; 7 Centro Salud San Blas, 28037 Madrid, Spain; ezequielarranz@gmail.com; 8 Specialist in Family and Community Medicine, Trujillo Health Center, 10200 Caceres, Spain; alfonso.barquilla@gmail.com; 9 Centro de Salud de A Estrada, 36680 Pontevedra, Spain; danielrey.aldana@usc.es; 10 Specialist in Family and Community Medicine, Casar de Caceres Health Center, 10190 Caceres, Spain; jpolog@semergen.es; 11 Networking Biomedical Research Centre-Cardiovascular Diseases (CIBERCV), 15782 Santiago de Compostela, Spain; scinzas@semergen.es

**Keywords:** blood pressure, cardiovascular, control, dapagliflozin, diabetes, glycated haemoglobin, weight

## Abstract

*Background and Objectives:* To assess the effects of adding dapagliflozin to an antidiabetic regimen, compared to standard of care (SOC) antidiabetic therapy and a cohort of patients who remained on a non-SGLT2i antidiabetic regimen without treatment modification despite persistent HbA1c elevation, on cardiometabolic control over a 12-month period in real-world settings. *Materials and Methods:* This ambispective (retrospective and prospective) observational study enrolled adults with type 2 diabetes who had received first-line metformin therapy, or an alternative antidiabetic agent in cases of intolerance or contraindication, excluding SGLT2 inhibitors, for a minimum of 12 months prior to recruitment. Patients were allocated to one of three groups: DAPA, SOC, or therapeutic inertia. Each patient attended three visits. The retrospective (V1–V0) and prospective (V0–V2) follow-up periods each extended for a minimum duration of six months. The primary endpoint was the change in cardiometabolic control from baseline to week 26 (V0), defined as HbA1c reduction ≥ 0.5%, weight loss ≥ 2 kg, and a systolic blood pressure drop ≥ 2 mmHg. *Results:* Five hundred thirty-five diabetes patients were included (39.1% women), with mean age (SD) 63.8 (8.2) years, body mass index 30.4 (4.9) kg/m^2^, and HbA1c 6.93 (0.9) %. More patients achieved cardiometabolic control with dapagliflozin (21.8%) vs. SOC + inertia (1.9%), OR 12.7 (95% CI 5.3–30.6), *p* < 0.001. The difference became greater over the entire study period. Event rates were low, but dapagliflozin exhibited fewer events numerically. The safety profile of dapagliflozin was consistent with previous findings. *Conclusions:* Treatment with dapagliflozin as add-on therapy was associated with improved cardiometabolic control over time compared to SOC, along with a numerical reduction in events.

## 1. Introduction

Type 2 diabetes mellitus (T2D) has reached epidemic levels, mainly due to obesity, sedentary habits, high calorie consumption, and an aging population [1,2]. In Spain, T2D has a prevalence of 15% among adults in primary care settings, with an age- and sex-adjusted prevalence of 11.5% [3].

Approximately half of patients are diagnosed with microvascular and macrovascular complications at the time of initial diagnosis [4]. Furthermore, the onset of heart failure and chronic kidney disease represents early complications observed in patients with T2D [5,6]. Attaining appropriate glycated haemoglobin A1c (HbA1c) control is essential for minimizing the risk of both microvascular and macrovascular complications [7]. Furthermore, clinical trials have demonstrated that sodium–glucose cotransporter-2 inhibitors (SGLT2i) provide cardiovascular and renal benefits beyond glycaemic control [8]. Traditional guidelines suggest starting most T2D patients on metformin, adding a second antidiabetic drug if HbA1c goals aren’t met, and introducing a third if needed for further control [9,10]. In recent years, for individuals with atherosclerotic cardiovascular disease, heart failure, or chronic kidney disease, medications with demonstrated cardiovascular or renal benefits should be considered as initial therapy [11]. Remarkably, optimal management of patients with T2D should prioritize not only glycaemic control but also a comprehensive strategy addressing multiple cardiovascular risk (CVR) factors, including hypertension, dyslipidemia or body weight (BW) [12].

Dapagliflozin was the first SGLT2i approved in Europe and holds the largest market share in Spain [13]. Various clinical trials (e.g., DECLARE-TIMI 58, DAPA-HF, DELIVER, DAPA-CKD) have reported that dapagliflozin is associated with positive effects on cardiovascular and renal outcomes, as well as changes in HbA1c levels, blood pressure, and BW in patients with T2D and different clinical conditions [14,15,16,17]. However, real-world data (RWD) is required to determine if the findings from clinical trials are applicable in clinical practice [18]. While some studies have confirmed these effects in clinical settings, most used a retrospective design [19].

The AGORA-AP study aimed to evaluate whether adding dapagliflozin to an antidiabetic regimen, as compared to standard of care (SOC) antidiabetic therapy, impacted cardiometabolic control over a 12-month period in real-world settings. The study also incorporated a separate cohort of patients who continued on a non-SGLT2i antidiabetic regimen without treatment modification despite persistent HbA1c elevation (designated as the inertia group) for analytical purposes.

## 2. Methods

**Figure 1:**
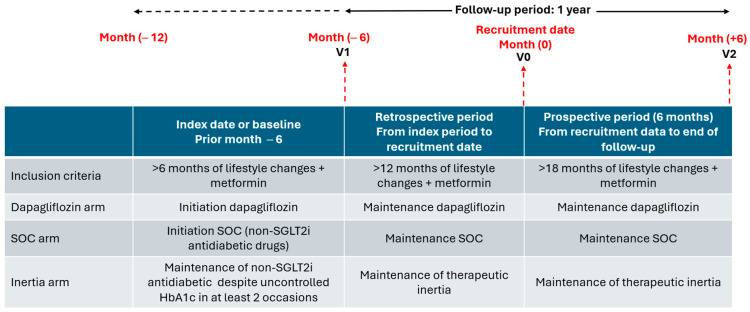
Study design. SGLT2i: sodium/glucose cotransporter 2 inhibitors; SOC: standard of care.

The methodology employed in this study has been detailed comprehensively in prior publications [20]. This was an ambispective observational longitudinal descriptive study (retrospective and prospective). A total of 61 primary care physicians from 46 primary care centres in the Spanish Health System consecutively recruited patients who met the inclusion criteria. To be included, adults aged 18–75 with T2D who, for more than 12 months before recruitment, were advised to quit smoking, lose at least 7% of their initial weight with healthy diets (low fat, high fibre), and do at least 150 min per week of aerobic exercise and strength training. Eligible participants were also required to be on first-line metformin therapy or another antidiabetic drug (excluding SGLT2i) if metformin was not tolerated or contraindicated, for at least 12 months prior to recruitment. Patients were assigned to one of the following treatment groups according to their antidiabetic regimen: (1) DAPA group: patients who began dapagliflozin treatment more than six months prior to recruitment; (2) SOC group: patients who started other antidiabetic drugs (excluding SGLT2i) more than six months prior to recruitment; (3) Inertia group: patients whose regimen with other antidiabetic drugs (excluding SGLT2i) was not changed or intensified, despite uncontrolled HbA1c in at least the last two assessments over more than six months before recruitment (Figure 1). The study excluded: patients with diabetes requiring insulin, GLP-1 receptor agonists, or SGLT2i (except dapagliflozin); those with type 1 diabetes or specific types such as monogenic diabetes, exocrine pancreas diseases, or drug-induced diabetes; pregnant, lactating, or gestational diabetes patients; individuals with creatinine > 2 mg/dL; those with uncorrected disabilities, moderate to severe cognitive impairment, dementia, schizophrenia or psychosis, residing in institutions, bedbound at home, or terminally ill; and anyone involved in other clinical studies or refusing participation. The study received approval from the Drug Research Ethics Committee of the Health Research Institute at the San Carlos University Clinical Hospital in Madrid, Spain, and was endorsed by the participating centres. The SEMERGEN foundation acted as the study promoter, and the Spanish Agency of Medicines and Medical Devices designated the research as a “post-authorization prospective follow-up study”. Patients gave written informed consent prior to inclusion.

The study consisted of three visits (Figure 1). V1 (index or baseline date): date on which study subjects initiated the oral antidiabetic drug treatment regimen (DAPA or SOC) or maintained therapeutic inertia. The same data obtained at the inclusion visit or recruitment were collected from the records of medical history close to the date on which treatment with the new antidiabetic drug (DAPA or SOC) was initiated or therapeutic inertia was maintained. V0 (inclusion or recruitment visit): at least 6 months after initiating the treatment regimen with oral antidiabetic drugs (DAPA or SOC) or maintenance of therapeutic inertia. The information relevant to the study was collected from the medical consultations with patients and from the records of the medical history. V2 (follow-up visit): 6 months after the date of inclusion or recruitment, maintaining the treatment regimen with oral antidiabetic drugs (dapagliflozin vs. SOC or therapeutic inertia). The same data from the index or baseline date plus changes in concomitant treatments were collected from medical consultations with patients and from the records of medical history. The retrospective analysis (minimum of 6 months) included the period between V1 and V0, and the prospective analysis between V0 and V2 (minimum of 6 months). The primary endpoint of the study was to compare in patients with T2DM in the second or third line of treatment (including metformin) in a routine clinical practice setting in Primary Care, the change experienced from baseline to week 26 (month 0) of the cardiometabolic control, measured by HbA1c reduction of at least 0.5%, BW loss of at least 2 kg, and systolic blood pressure (SBP) decrease of at least 2 mmHg, produced by dapagliflozin versus the cardiometabolic control produced by other non-SGLT2i oral antidiabetic drugs.

The collected variables encompassed socio-demographic information, findings from physical examinations, CVR factors, evidence of target organ damage, presence of cardiovascular disease, relevant biochemical parameters, and details regarding concomitant treatments including antidiabetic medications, antihypertensive agents, and lipid-lowering therapies. The definitions of variables, targets, and approaches to CVR stratification were established in accordance with the 2016 European Guidelines on cardiovascular disease prevention in clinical practice, as subsequently endorsed by the 2021 European Guidelines [21,22]. Alcoholism was defined as consuming more than 21 standard drinks per week for men, and more than 14 for women (1 unit = 10 g alcohol). Obesity was defined as a body mass index (BMI) of 30 kg/m^2^ or higher, while central obesity as an increased waist circumference (≥102 cm for men, ≥88 cm for women). Adiposity or body fat index CUN-BAE (Clínica Universitaria de Navarra—Body Adiposity Estimator) was defined as −44.988 + (0.503 × age) + (3.172 × BMI) − (0.026 × BMI^2^) − (0.02 × BMI × age) + (0.00021 × BMI^2^ × age) in men, and as −44.988 + (0.503 × age) + 10.689 + (3.172 × BMI) − (0.026 × BMI^2^) + (0.181 × BMI) − (0.02 × BMI × age) − (0.005 × BMI^2^) + (0.00021 × BMI^2^ × age) in women. Adiposity, measured by CUN-BAE, was defined as >25% for males and >35% for females [23]. A waist-to-height ratio of 0.55 or higher was classified as high [24]. A fatty liver index below 30 effectively excluded the presence of steatosis liver disease, while an index of 60 or higher supported its diagnosis [25]. Major adverse cardiovascular events (MACE) was defined as a 3-point outcome comprising cardiovascular death, nonfatal myocardial infarction, or nonfatal stroke. A low eGFR was specified as <60 mL/min/1.73 m^2^ according to CKD-EPI equations, and albuminuria was defined as a urine albumin-creatinine ratio (uACR) ≥ 30 mg/g [26].

Differences in biochemical parameters, anthropometric data, and the achievement of cardiometabolic control targets (HbA1c, BW, SBP, and/or fasting plasma glucose) between groups were assessed during the study period. Incidences of comorbidities (such as dyslipidemia, hypertension, atrial fibrillation, heart failure, hospitalization for heart failure, myocardial infarction, stroke, peripheral artery disease, all-cause death, cardiovascular death, and MACE) and adverse events between V1 and V2 were recorded.

### Statistical Analysis

Qualitative variables were summarized by number and percentage and compared using Chi-square or Fisher’s exact tests. Continuous variables were analysed with mean and standard deviation (SD), and compared via Student’s *t*, Levene, and ANOVA tests. Cohen’s *d* measured effect size between group means (<0.2 low; 0.5 moderate; 0.8 high; 1.3 very high). Odds ratios (with 95% confidence intervals) were calculated to assess associations in cardiometabolic control targets between groups. Additionally, the incidence density per 1000 patient-years and rate ratios were calculated for adverse clinical events. To make the groups comparable, the main results of the study were adjusted for age, residence setting, education level, and employment status. Missing data were managed with simple imputation, replacing missing values with the mean or median of the observed data for that variable. All analyses were two-sided, with significance at *p* <0.05. Statistical analysis used SPSS 23.0 and/or STATA 11.0.

## 3. Results

Between April 2018 and June 2023, 783 subjects with T2D were recruited. Of these, 605 met all inclusion and exclusion criteria. Ultimately, 535 subjects completed all three visits and were included in the final analysis. The main reason it was decided to start DAPA or SOC was poor HbA1c control, followed by improving HbA1c control and obesity, without significant differences between groups (Supplementary Table S1)

Baseline, the mean age of the patients (SD) was 63.8 (8.2) years, 39.1% were women, the BMI was 30.4 (4.9) kg/m^2^, and the SBP was 131.6 (13.7) mm Hg. The presence of CVR factors was frequent, with dyslipidemia (80.6%) and hypertension (70.5%) being the most common. 16.2% had coronary artery disease and 11.9% prior stroke. HbA1c was 6.93 (0.9) %. 43.2% had a high CVR, 45.6% very high CVR and 11.2% an extreme CVR. If baseline characteristics are compared between the treatment arms, the DAPA group was slightly younger (*p* = 0.002), had a higher BMI (*p* = 0.04), a higher proportion of patients with a very high CVR (*p* = 0.001), a higher HbA1c (*p* < 0.001) and less microalbuminuria (*p* = 0.012), with no significant differences in the rest of the clinical features (Table 1). Obesity was present in 48.8% and abdominal obesity in 73.6% of individuals, without significant differences between groups (Supplementary Table S2). Of the participants, 10.1% used other antidiabetic drugs (higher in the inertia arm), 67.1% took antihypertensives drugs, and 80.4% used lipid-lowering agents (higher in the DAPA arm) (Supplementary Table S3). No significant differences in drug variation were found between groups (Supplementary Table S4).

**Figure 2:**
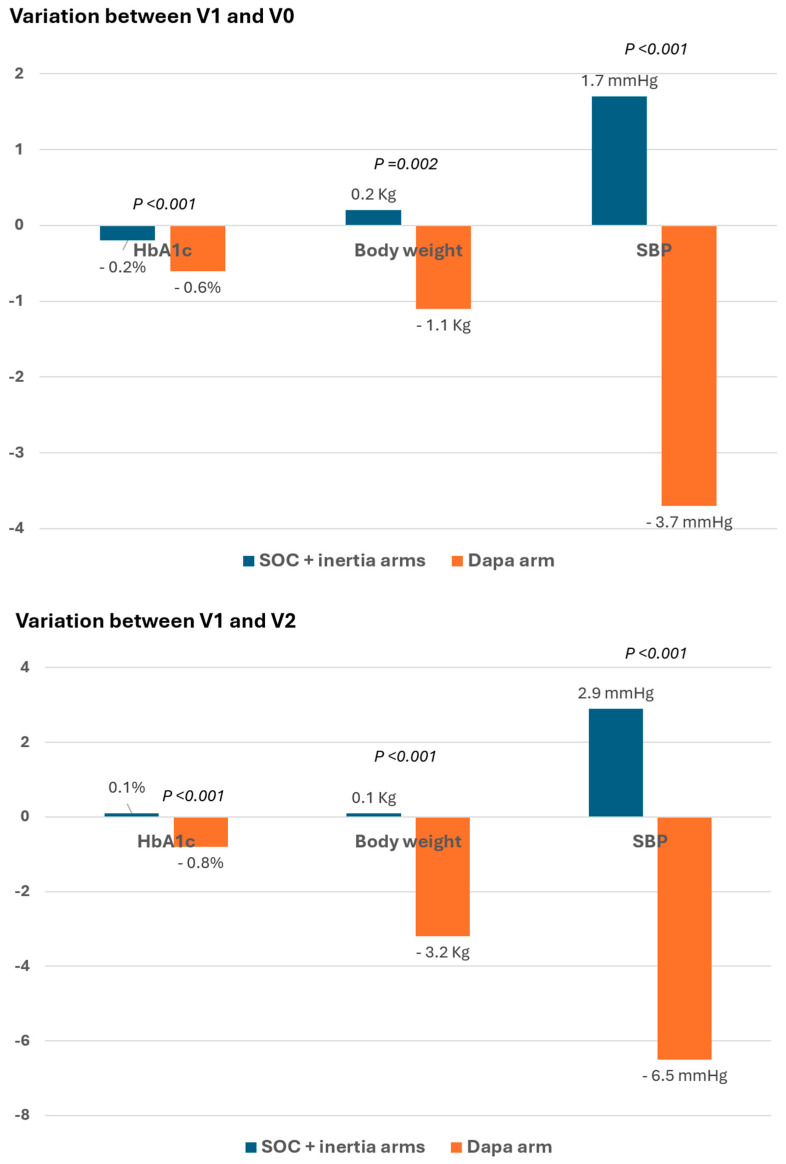
Changes in HbA1c, body weight and SBP between groups during the study period. Dapa: dapagliflozin; HbA1c: glycated hemoglobin A1c; SOC: standard of care; SBP: systolic blood pressure.

Anthropometric data changes (Figure 2 and Supplementary Table S5) showed greater reductions in BW, waist circumference, waist-to-height ratio, and SBP in the DAPA arm compared to SOC + inertia arms from V1 to V0, with these differences becoming more pronounced between V0 and V2. Fasting plasma glucose, HbA1c, uric acid, and albuminuria decreased more in the DAPA group than in SOC and inertia groups from V1 to V0, with greater differences observed from V0 to V2 (Figure 2 and Supplementary Table S6).

The prevalence and degree of control of the main biochemical parameters are shown in Supplementary Table S7. More patients achieved cardiometabolic control (HbA1c reduction ≥ 0.5%, BW loss ≥ 2 kg, SBP decrease ≥ 2 mm Hg) with dapagliflozin (21.8%) than with other non-SGLT2i oral antidiabetic drugs (1.9%; OR = 12.7, *p* < 0.001), after adjusting for covariables. The difference became greater over the entire study period. Additionally, patients in the DAPA group achieved cardiometabolic control in a much higher proportion than patients in the therapeutic inertia arm. By contrast, a similar proportion of patients in the SOC arm and those with therapeutic inertia achieved cardiometabolic control (Table 2). On the other hand, across all HbA1c thresholds, the proportion of patients achieving glycaemic control at V0 was lower in the DAPA arm compared to the SOC or inertia arm, with this disparity increasing as control targets became more rigorous. At V2, however, the percentages of patients attaining good control at various HbA1c levels were marginally higher in the DAPA arm than in the SOC or inertia arm, except for the strictest target (HbA1c < 6.5%), where this trend did not persist (Supplementary Table S8). Furthermore, patients in the DAPA and SOC or inertia arms achieved a reduction in fasting plasma glucose in a similar proportion (supplementary Table S9). Additionally, patients in the DAPA arm achieved the combined reduction in HbA1c and SBP in a much higher proportion than patients in the SOC or inertia group, which increased throughout the study. Patients in the DAPA arm achieved a greater combined reduction in HbA1c and BW than patients in the SOC or inertia group, which increased throughout the study. Finally, patients in the DAPA arm achieved a greater proportion of combined BW and SBP reduction than patients in the SOC or inertia group, which was maintained throughout the study (Supplementary Table S9). The incidence of events and comorbidities observed during the study was specifically evaluated (Table 3). Overall, event rates were low, and no significant differences were found between groups. The DAPA arm had numerically fewer adverse events than the SOC or inertia group, except for a higher rate of genital infections (Supplementary Table S10).

## 4. Discussion

In a large cohort, our study found that obesity and additional CVR factors were prevalent among patients with T2D, resulting in a substantial proportion of individuals being classified as high- or very high-CVR. Patients receiving dapagliflozin demonstrated superior cardiometabolic control over time compared to those managed with SOC. Although overall event rates were low, the dapagliflozin group experienced fewer adverse events numerically than the SOC arm. Apart from an increased incidence of genital infections, side effect rates did not differ significantly between the treatment groups.

Since T2D is progressive, providing adequate management to delay the development and progression of complications seems mandatory. In this context, maintaining HbA1c targets typically requires timely combination therapy that includes agents with complementary mechanisms of action [27]. Strict and early control of HbA1c provides both macrovascular and microvascular benefits, particularly when levels are reduced to below 7% [11]. According to the ADA 2025 recommendations for T2D management, early combination of antidiabetic therapy in addition to healthy lifestyle habits is key to achieving recommended HbA1c targets [11,27]. Additionally, the guidelines also emphasize the critical importance of addressing other relevant factors to reduce CVR [12]. Dapagliflozin is an SGLT2i that is commonly used in Spain [28]. Therefore, it is important to evaluate its impact on cardiometabolic control and clinical events in actual patients. This study presents data relevant to this assessment. Furthermore, the ambispective study design is valuable because it offers more insight than a purely retrospective or prospective approach. While our study is observational and non-interventional, prospective follow-up may still affect patient behaviour. Therefore, collecting both retrospective and prospective data is essential, thereby enhancing the robustness of our findings.

The study population had a mean age of 64 years; 39% were women, BMI averaged 30 kg/m^2^, mean HbA1c was 6.9%, and patients had a high, very high, or even extreme level of CVR. In the DECLARE-TIMI 58 trial, that included patients with T2D who had or were at risk for atherosclerotic cardiovascular disease, mean age was 64 years, 37% were women, BMI averaged 32 kg/m^2^, mean HbA1c was 8.3%, and 33% had coronary artery disease [14]. A retrospective cohort study reported a mean age of 58 years, with 41% women, a mean BMI of 35 kg/m^2^, and 14% with ischemic heart disease in patients with diabetes [19]. In Spain, a cross-sectional study in Primary Care found that among patients with diabetes mean age was 67 years, BMI 30.0 kg/m^2^, 5.5%, 9%, 74%, and 12% of these patients had moderate, high, very high, and extreme CVR, respectively [3]. Despite differences in the study design and inclusion criteria, data from clinical trials and RWD show that patients with T2D have many comorbidities, such as obesity and have high CVR. This makes the management of these patients more challenging. Overall, the groups showed similar clinical characteristics, with only minor differences in BMI and HbA1c, both higher in the DAPA group. Since this was an RWD study, physicians likely added dapagliflozin before enrolment for T2D patients with higher BW and poor HbA1c control, due to its known effects on these factors [29].

No significant differences in drug variation were found between groups, indicating that changes in biochemical and anthropometric measures were linked to dapagliflozin treatment versus SOC. Dapagliflozin led to greater reductions in BW, waist circumference, waist-to-height ratio, SBP, fasting plasma glucose, and HbA1c than SOC or inertia group, with differences increasing over time. Consequently, a higher proportion of patients in the dapagliflozin group achieved complete cardiometabolic control. This difference became more pronounced throughout the study period and was especially notable compared to the inertia group. By contrast, an equivalent proportion of patients in the SOC arm and those experiencing therapeutic inertia reached cardiometabolic control. On the other hand, although fewer patients in the dapagliflozin group achieved glycaemic control during the retrospective study phase compared to the SOC or inertia arm, this pattern later reversed. Our data were consistent with previous studies that indicate that dapagliflozin offers moderate to high HbA1c reduction, can be safely combined with metformin or other T2D therapies, and is linked to moderate weight loss, notable SBP reductions, and important cardiovascular and renal benefits [29,30]. In the DECLARE-TIMI 58 trial, participants receiving dapagliflozin demonstrated consistently lower HbA1c levels compared to those in the placebo group, with a mean absolute difference of 0.42%. Furthermore, the dapagliflozin group experienced a mean reduction in BW of 1.8 kg and a decrease in SBP of 2.7 mm Hg relative to placebo [14]. Remarkably, our results were also consistent with findings from real-life studies [31,32,33]. In summary, SGLT2i provide benefits beyond glucose lowering, indicating additional complex and multifactorial mechanisms are involved. Thus, evidence indicates that SGLT2i enhances cardiometabolic outcomes by lowering SBP, reducing BW and BMI, improving glycemic control, and decreasing triglyceride levels. Additionally, these drugs directly benefit the cardiovascular system by lowering inflammation, oxidative stress, and arterial stiffness while enhancing cardiac energy metabolism and function. These agents also affect the nervous system by suppressing sympathetic activity and may facilitate the utilization of fat as an energy source. More recently, it has been suggested that a reduction in sudden cardiac death risk with SGLT2i [34,35,36]. Given the high prevalence of comorbidities such as obesity and hypertension among patients with T2D, achieving comprehensive cardiometabolic control, rather than focusing solely on HbA1c, underscores the support and added value offered by treatment with dapagliflozin.

Clinical trials have demonstrated that dapagliflozin reduces cardiovascular and renal outcomes in individuals with T2D, with notable effects observed among those with heart failure and chronic kidney disease at baseline [14,15,16,17]. Our study found no significant decrease in events and comorbidities, likely due to insufficient sample size. Nevertheless, a notable decrease in albuminuria was observed with dapagliflozin administration. Moreover, our findings align with clinical trials. Additionally, some RWD studies have reported fewer events, including mortality, in T2D patients treated with dapagliflozin, regardless of baseline cardiovascular disease status [19].

Dapagliflozin treatment was associated with fewer adverse events compared to the SOC plus inertia group, although it resulted in a higher incidence of genital infections. In the DECLARE-TIMI 58 trial, a lower proportion of participants in the dapagliflozin group discontinued their assigned regimen compared to those in the placebo group. Additionally, serious adverse events, including major hypoglycaemia, acute kidney injury, or bladder cancer, were less frequently reported among patients receiving dapagliflozin. The incidence of amputation, fracture, and volume depletion was similar across both groups. As anticipated, the occurrence of genital infections was higher in the dapagliflozin group; however, reports of genital infections as serious adverse events remained infrequent [14]. As a result, our findings were consistent with those of the DECLARE-TIMI 58 trial.

This study presents the typical limitations associated with observational research. The analysis of non-comparable groups of patients starting dapagliflozin or other oral antidiabetic therapies may result in differences in certain clinical characteristics, which could introduce some confounding results. Furthermore, adherence to medication was not determined. Additionally, patients with advanced renal disease were excluded due to the health authority requirements at the time the study protocol was authorized. Although we chose dapagliflozin as a comparator drug, since it is the most frequently prescribed SGLT-2i in the primary care setting, this could have introduced a potential bias. Lastly, the results can only be extrapolated to other countries and health care systems with patients with a clinical profile similar to that of Spain.

In summary, treatment with dapagliflozin as add-on therapy in patients with T2D was associated with improved cardiometabolic control over time compared to those managed with SOC, along with a numerical reduction in events and a safety profile consistent with previous findings. These results suggest that dapagliflozin should be considered an option for the management of patients with T2D in primary care settings.

## Figures and Tables

**Table 1:** Baseline clinical characteristics of the study population.

	Dapa Arm (n = 226)	SOC Arm (n = 197)	Inertia Arm (n = 112)	Total (n = 535)	*p*
Socio-demographic data					
Age, years	62.3 (7.9)	64.9 (8.2)	64.6 (8.2)	63.8 (8.2)	0.002
Sex (women), n (%)	90 (39.8)	72 (36.5)	47 (42.0)	209 (39.1)	0.614
Residence setting, n (%)					0.003
Urban (>20,000 inhabitants)	178 (78.8)	153 (77.7)	82 (73.2)	413 (77.2)	
Semi-urban (5000 to 20,000 inhabitants)	24 (10.6)	12 (6.1)	3 (2.7)	39 (7.3)	
Rural (<5000 inhabitants)	24 (10.6)	32 (16.2)	27 (24.1)	83 (15.5)	
Race/ethnicity, n (%)					0.29
White	218 (96.5)	190 (96.4)	106 (94.6)	514 (96.1)	
Black	4 (1.8)	0 (0)	1 (0.9)	5 (0.9)	
Latin American	2 (0.9)	4 (2.0)	1 (0.9)	7 (1.3)	
Asian	0 (0)	1 (0.5)	2 (1.8)	3 (0.6)	
Maghrebi/Arab	2 (0.9)	2 (1.0)	2 (1.8)	6 (1.1)	
Education level, n (%)					<0.001
Without studies	11 (4.9)	13 (6.6)	6 (5.4)	30 (5.6)	
Primary studies	121 (53.5)	108 (54.8)	80 (71.4)	309 (57.8)	
Higher studies	77 (34.1)	66 (33.5)	18 (16.1)	161 (30.1)	
University studies	17 (7.5)	10 (5.1)	4 (3.6)	31 (5.8)	
Housewife	0 (0)	0 (0)	4 (3.6)	4 (0.7)	
Employment status, n (%)					0.017
Employed	94 (41.6)	66 (33.5)	32 (28.6)	192 (35.9)	
Unemployed	7 (3.1)	2 (1.0)	5 (4.5)	14 (2.6)	
Retired/pensioner	96 (42.5)	111 (56.3)	56 (50.0)	263 (49.2)	
Homework	29 (12.8)	18 (9.1)	19 (17.0)	66 (12.3)	
Alcohol consumption, n (%)	28 (12.4)	25 (12.7)	14 (12.5)	67 (12.5)	0.996
Physical examination					
BMI, Kg/m^2^	31.0 (5.1)	29.8 (4.6)	30.1 (4.6)	30.4 (4.9)	0.040
Body weight, Kg	84.9 (16.2)	81.7 (15.6)	83.5 (16.4)	83.4 (16.0)	0.114
Waist-to-height ratio	0.63 (0.06)	0.63 (0.07)	0.64 (0.08)	0.63 (0.07)	0.601
Waist circumference, cm	104.3 (10.2)	103.3 (10.6)	104.0 (12.7)	103.9 (10.8)	0.717
Percentage of body fat CUNBAE (%), mean (SD)	38.0 (7.9)	36.0 (7.3)	38.0 (8.1)	37.3 (7.8)	0.035
SBP, mmHg	129.3 (12.7)	133.2 (13.4)	133.5 (15.5)	131.6 (13.7)	0.004
DBP, mmHg	77.7 (8.8)	78.5 (9.2)	77.2 (8.8)	77.9 (8.9)	0.391
Pulse pressure, mmHg	51.6 (10.2)	54.8 (10.6)	55.4 (13.3)	53.5 (11.2)	0.002
Heart rate, bpm	71.6 (10.1)	73.4 (10.3)	73.7 (10.7)	72.7 (10.3)	0.113
CVR factors					
Dyslipidemia, n (%)	192 (85.0)	157 (79.7)	82 (73.2)	431 (80.6)	0.034
Important dyslipidemia, n (%)	7 (3.1)	2 (1.0)	2 (1.8)	11 (2.1)	0.314
Hypertension, n (%)	160 (70.8)	137 (69.5)	80 (71.4)	377 (70.5)	0.931
Overweight (BMI 25 to 29.99 Kg/m^2^), n (%)	88 (39.1)	89 (45.4)	41 (36.6)	218 (40.9)	0.237
Obesity (BMI ≥ 30 Kg/m^2^), n (%)	118 (52.4)	87 (44.4)	55 (49.1)	260 (48.8)	
Sedentarism, n (%)	96 (42.5)	92 (46.7)	57 (50.9)	245 (45.8)	0.326
Smoking, n (%)					0.081
Current	48 (21.2)	37 (18.8)	12 (10.7)	97 (18.1)	
Former	72 (31.9)	61 (31.0)	31 (27.7)	164 (30.7)	
Never	106 (46.9)	99 (50.3)	69 (61.6)	274 (51.2)	
Target organ damage					
Eye fundus examination performed, n (%)	163 (72.1)	132 (67.0)	62 (55.4)	357 (66.7)	0.009
LVH, n (%)	9 (4.0)	10 (5.1)	9 (8.0)	28 (5.2)	0.287
ABI < 0.9, n (%)	13 (5.8)	6 (3.0)	7 (6.3)	26 (4.9)	0.323
Carotid atherosclerotic plaques, n (%)	6 (2.7)	6 (3.0)	7 (6.3)	19 (3.6)	0.217
Increased carotid Intima-media thickness, n (%)	6 (2.7)	5 (2.5)	6 (5.4)	17 (3.2)	0.334
CV disease					
Coronary artery disease, n (%)	19 (18.8)	12 (11.7)	11 (20.0)	42 (16.2)	0.264
Stroke, n (%)	9 (8.9)	16 (15.4)	6 (10.9)	31 (11.9)	0.348
Albuminuria (30–299 mg/g), n (%)	23 (10.2)	21 (10.7)	15 (13.4)	59 (11.0)	0.660
Peripheral artery disease, n (%)	11 (11.1)	11 (10.7)	3 (6.0)	25 (9.9)	0.582
CKD (eGFR < 60 mL/min), n (%)	7 (3.1)	1 (0.5)	4 (3.6)	12 (2.2)	0.113
Proteinuria (≤300 mg/g), n (%)	4 (1.8)	1 (0.5)	3 (2.7)	8 (1.5)	0.289
Heart failure, n (%)	5 (2.2)	3 (1.5)	0 (0)	8 (1.5)	0.288
CVR score, n (%)					0.001
High	83 (36.7)	82 (41.6)	66 (58.9)	231 (43.2)	
Very high	119 (52.7)	87 (44.2)	38 (33.9)	244 (45.6)	
Extreme	24 (10.6)	28 (14.2)	8 (7.1)	60 (11.2)	
Biochemical parameters					
Hemoglobin (g/dL), mean (SD)	15.2 (1.5)	14.4 (1.2)	14.7 (1.3)	14.8 (1.4)	<0.001
FPG (mg/dL), mean (SD)	138.6 (33.8)	133.5 (32.7)	134.3 (29.3)	135.8 (32.5)	0.242
HbA1c (%), mean (SD)	7.1 (0.9)	6.7 (0.8)	7.0 (0.9)	6.93 (0.9)	<0.001
TC (mg/dL), mean (SD)	168.3 (35.8)	169.0 (36.0)	171.5 (33.3)	169.2 (35.4)	0.73
HDL-c (mg/dL), mean (SD)	47.7 (11.4)	48.7 (11.1)	47.5 (12.1)	48.0 (11.4)	0.587
LDL-c (mg/dL), mean (SD)	90.1 (31.7)	90.5 (31.3)	93.1 (29.2)	90.9 (31.0)	0.7
TG (mg/dL), mean (SD)	156.6 (66.3)	145.7 (81.2)	149.7 (68.8)	151.1 (72.6)	0.299
Sodium (mEq/L), mean (SD)	140.8 (2.2)	140.4 (2.8)	140.8 (2.7)	140.6 (2.5)	0.314
Potassium (mEq/L), mean (SD)	4.6 (0.4)	4.6 (0.4)	4.6 (0.4)	4.6 (0.4)	0.338
SUA (mg/dL), mean (SD)	4.9 (1.2)	5.5 (1.2)	5.5 (1.4)	5.2 (1.3)	<0.001
AST (U/L), mean (SD)	26.4 (13.0)	25.0 (14.0)	26.5 (13.0)	25.9 (13.4)	0.702
ALT (U/L), mean (SD)	28.2 (17.6)	24.7 (13.1)	27.0 (14.8)	26.7 (15.6)	0.087
GGT (U/L), mean (SD)	30.9 (19.5)	34.7 (24.7)	31.3 (20.1)	32.3 (21.6)	0.247
FLI, mean (SD)	69.8 (21.3)	66.7 (23.1)	66.9 (26.1)	68.2 (22.9)	0.46
Creatinine (mg/dL), mean (SD)	0.8 (0.2)	0.9 (0.2)	0.9 (0.2)	0.8 (0.2)	0.106
eGFR (mL/min/1.73 m^2^), mean (SD)	99.2 (16.3)	96.7 (15.9)	96.6 (18.9)	97.7 (16.7)	0.23
uACR (mg/g), mean (SD)	13.3 (12.8)	15.3 (15.2)	19.6 (21.0)	15.2 (15.6)	0.012

ABI: ankle brachial index; ALT: alanine aminotransferase; AST: aspartate aminotransferase; BMI: body mass index; CKD: chronic kidney disease; CV: cardiovascular; CVR: cardiovascular risk; Dapa: dapagliflozin; DBP: diastolic blood pressure; eGFR: estimated glomerular filtration rate; FPG: fasting plasma glucose; FLI: fatty liver index; GGT: gamma-glutamyl transferase; HbA1c: glycated hemoglobin A1c; HDL-c: high-density lipoprotein cholesterol; LDL-c: low-density lipoprotein cholesterol; LVH: left ventricular hypertrophy; SOC: standard of care; SUA: serum uric acid; TC: total cholesterol; SBP: systolic blood pressure; TG: triglycerides; uACR: urine albumin-creatinine ratio.

**Table 2:** Achievement of the main cardiometabolic control objective (HbA1c, body weight and SBP) between groups during the study period.

Reduction HbA1c ≥ 0.5% + reduction body weight ≥ 2 kg + reduction SBP ≥ 2 mmHg between V1 and V0		Adjusted for age, residence setting, education level, employment status	
		OR (95% CI)	*p*
SOC + Inertia arms, n (%)	6 (1.9)	1 Reference	<0.001
DAPA arm, n (%)	49 (21.8)	12.7 (5.3–30.6)	
Reduction HbA1c ≥ 0.5% + reduction body weight ≥ 2 kg + reduction SBP ≥ 2 mmHg between V1 and V2		Adjusted for age, residence setting, education level, employment status	
		OR (95% CI)	*p*
SOC + Inertia arms, n (%)	12 (4.0)	1 Reference	<0.001
DAPA arm, n (%)	82 (37.3)	14.4 (7.5–27.7)	
Reduction HbA1c ≥ 0.5% + reduction body weight ≥ 2 kg + reduction SBP ≥ 2 mmHg between V1 and V0		Adjusted for age, residence setting, education level, employment status	
		OR (95% CI)	*p*
Inertia arm, n (%)	2 (1.8)	1 Reference	0.001
DAPA arm, n (%)	49 (21.8)	13.3 (3.1–57.5)	
Reduction HbA1c ≥ 0.5% + reduction body weight ≥ 2 kg + reduction SBP ≥ 2 mmHg between V1 and V2		Adjusted for age, residence setting, education level, employment status	
		OR (95% CI)	*p*
Inertia arm, n (%)	5 (4.5)	1 Reference	<0.001
DAPA arm, n (%)	82 (37.3)	12.0 (4.6–31.5)	
Reduction HbA1c ≥ 0.5% + reduction body weight ≥ 2 kg + reduction SBP ≥ 2 mmHg between V1 and V0		Adjusted for age, residence setting, education level, employment status	
		OR (95% CI)	*p*
Inertia arm, n (%)	2 (1.8)	1 Reference	0.983
SOC arm, n (%)	4 (2.0)	1.0 (0.2–6.2)	
Reduction HbA1c ≥ 0.5% + reduction body weight ≥ 2 kg + reduction SBP ≥ 2 mmHg between V1 and V2		Adjusted for age, residence setting, education level, employment status	
		OR (95% CI)	*p*
Inertia arm, n (%)	5 (4.5)	1 Reference	0.523
SOC arm, n (%)	7 (3.6)	0.7 (0.2–2.3)	

Dapa: dapagliflozin; HbA1c: glycated hemoglobin A1c; SOC: standard of care; OR: Odds Ratio; SBP: systolic blood pressure: 95% CI: 95% confidence interval.

**Table 3:** Incidence of events and comorbidities between V1 and V2.

	SOC + Inertia Arms		Dapa Arm		Adjusted for Age, Residence Setting, Education Level, Employment Status			
	n (%)	Incidence Density Per 1000 Patient-Years	n (%)	Incidence Density Per 1000 Patient-Years	OR (95% CI)	*p*	Rate Ratio (95% CI)	*p*
Incidence of dyslipidemia	12 (4.3)		7 (3.3)		0.6 (0.2–1.6)	0.286		
Incidence of hypertension	15 (5.7)		11 (6.0)		0.9 (0.4–2.1)	0.791		
Incidence of atrial fibrillation	8 (2.6)	4.8	7 (3.1)	6.5	1.2 (0.4–3.5)	0.769	1.4 (0.4–4.3)	0.122
Incidence of heart failure	1 (0.3)	0.6	2 (0.9)	1.8	3.5 (0.2–49.9)	0.363	3.1 (0.2–180.6)	0.448
Incidence of heart failure hospitalization	0 (0.0)		0 (0.0)		NC			
Incidence of myocardial infarction	4 (1.3)	2.4	2 (0.9)	1.8	0.7 (0.1–4.2)	0.705	0.8 (0.07–5.3)	0.098
Incidence of stroke	8 (2.6)	4.9	1 (0.4)	0.9	0.2 (0.03–1.9)	0.178	0.2 (0.004–1.4)	0.32
Incidence of peripheral artery disease	3 (1.0)	1.8	3 (1.3)	2.7	1.2 (0.2–6.5)	0.803	1.5 (0.2–11.5)	0.17
Death (V2)	2 (0.6)	1.2	3 (1.3)	2.7	2.7 (0.4–18.4)	0.3	2.3 (0.3–27.5)	0.34
Cardiovascular death (V2)	0 (0.0)		0 (0.0)		NC			
Incidence of MACE	10 (3.2)	6.0	3 (1.3)	2.7	0.5 (0.1–1.8)	0.275	0.5 (0.08–1.8)	0.21

NC: not computable because there were no cases in any of the groups. Incident event or comorbidity: new cases that did not exist in V1 and have appeared in the V1-V2 period. Dapa: dapagliflozin; OR: Odds Ratio; MACE: major adverse cardiovascular events; SOC: standard of care; 95% CI: 95% confidence interval.

## Data Availability

All data concerning the case are presented in this manuscript.

## Supplementary Materials

The following supporting information can be downloaded at: https://www.mdpi.com/article/10.3390/medicina61122087/s1, Table S1: Reasons why it was decided to initiate or discontinue treatment with dapagliflozin or with other non-SGLT-2i antidiabetic drugs; Table S2: Prevalence of obesity at baseline; Table S3: Baseline treatments at the time of recruitment (V0); Table S4: Changes in concomitant treatments between groups during the study period; Table S5: Changes in anthropometric data between groups during the study period; Table S6: Changes in biochemical parameters between groups during the study period; Table S7: Prevalence and degree of control of the main biochemical parameters; Table S8: Achievement of the goal of reaching HbA1c levels < 8.0%, <7.5%, <7.0% and <6.5% between groups in the different study visits; Table S9: Achievement of the different cardiometabolic control objectives (HbA1c, body weight, SBP and/or FPG) between groups during the study period; Table S10: Incidence of adverse events between V1 and V2.
