# Supplementary material for: Improvement of Cardiometabolic Control with Dapagliflozin in Patients with Type 2 Diabetes in Primary Care: The AGORA-AP Study
Source: Medicina (Kaunas). 2025 Nov 23;61(12):2087. doi: 10.3390/medicina61122087 (PMC12734714; doi:10.3390/medicina61122087)
Supplement: Supplementary file 1 [file medicina-61-02087-s001.zip › medicina-3971391-supplementary.pdf]

Supplementary Table S1. Reasons why it was decided to initiate or discontinue treatment with dapagliflozin or with other non-SGLT-2i antidiabetic drugs.

Reasons why it was decided to initiate dapa or SOC in V1	SOC + inertia arms	Dapa arm	P
Poor HbA1c control, n (%)	85 (43.1)	117 (51.8)	0.118
Improve HbA1c, n (%)	56 (28.4)	56 (24.8)	
Obesity, n (%)	43 (21.8)	47 (20.8)	
Others, n (%)	13 (6.6)	6 (2.7)	

Dapa: dapagliflozin; HbA1c: glycated hemoglobin A1c; SOC: standard of care.

Supplementary Table S2. Prevalence of obesity at baseline.

	Definition	Dapa arm (n=226)	SOC arm (n=197)	Inertia arm (n=112)	Total (n=535)	P
Obesity BMI SEEDO	Underweight (<18.5 Kg/m^2), n (%)	0 (0.0)	1 (0.5)	0 (0.0)	1 (0.2)	0.318
	Normal weight (18.5 to 24.99 Kg/m^2), n (%)	19 (8.4)	19 (9.7)	16 (14.3)	54 (10.1)	
	Grade I overweight (25 to 26.99 Kg/m^2), n (%)	32 (14.2)	39 (19.9)	17 (15.2)	88 (16.5)	
	Grade II overweight (27 a 29.99 Kg/m^2), n (%)	56 (24.9)	50 (25.5)	24 (21.4)	130 (24.4)	
	Type I obesity (30 to 34.99 Kg/m^2), n (%)	75 (33.3)	58 (29.6)	40 (35.7)	173 (32.5)	
	Type II obesity (35 to 39.99 Kg/m^2), n (%)	29 (12.9)	22 (11.2)	13 (11.6)	64 (12.0)	
	Type III obesity (40 to 49.99 Kg/m^2), n (%)	14 (6.2)	6 (3.1)	1 (0.9)	21 (3.9)	
	Type IV obesity (≥50 Kg/m^2), n (%)	0 (0.0)	1 (0.5)	1 (0.9)	2 (0.4)	
Obesity BMI	Normal weight (≤24.99 Kg/m^2), n (%)	19 (8.4)	20 (10.2)	16 (14.3)	55 (10.3)	0.237
	Overweight (25 to 29.99 Kg/m^2), n (%)	88 (39.1)	89 (45.4)	41 (36.6)	218 (40.9)	
	Obesity (≥30 Kg/m^2), n (%)	118 (52.4)	87 (44.4)	55 (49.1)	260 (48.8)	
Abdominal obesity	Men ≥102 cm; Women: ≥88 cm, n (%)	131 (73.6)	103 (70.1)	58 (80.6)	292 (73.6)	0.255
Waist-to-height ratio	>0.5, n (%)	178 (100)	145 (98.6)	70 (97.2)	393 (99.0)	
Obesity CUNBAE	Men:>25%; Women: >35%, n (%)	221 (97.8)	191 (97.0)	109 (97.3)	521 (97.4)	0.865

BMI: body mass index; dapa: dapagliflozin; SEEDO: Sociedad Española para el Estudio de la Obesidad (Spanish Society for the Study of Obesity); SOC: standard of care.

Supplementary Table S3. Baseline treatments at the time of recruitment (V0).

	Dapa arm (n=226)	SOC arm (n=197)	Inertia arm (n=112)	Total (n=535)	P
**Other antidiabetic drugs, (%)**	20 (8.8)	9 (4.6)	25 (22.3)	54 (10.1)	<0.001
Pioglitazone, (%)	0 (0.0)	1 (0.5)	2 (1.8)	3 (0.6)	0.117
Alpha glucosidase inhibitor, (%)	0 (0.0)	0 (0.0)	0 (0.0)	0 (0.0)	--
Other hypoglycemic agents, (%)	20 (8.8)	8 (4.1)	24 (21.4)	52 (9.7)	<0.001
**Antihypertensive drugs, (%)**	150 (66.4)	130 (66.0)	79 (70.5)	359 (67.1)	0.683
Diuretics, (%)	74 (32.7)	67 (34.0)	34 (30.4)	175 (32.7)	0.805
ACEIs, (%)	84 (37.2)	67 (34.0)	38 (33.9)	189 (35.3)	0.748
ARBs, (%)	56 (24.8)	54 (27.4)	36 (32.1)	146 (27.3)	0.359
Calcium channel blockers, (%)	36 (15.9)	40 (20.3)	19 (17.0)	95 (17.8)	0.487
Beta blockers, (%)	17 (7.5)	22 (11.2)	10 (8.9)	49 (9.2)	0.43
Alpha blockers, (%)	3 (1.3)	3 (1.5)	7 (6.3)	13 (2.4)	0.013
Centrally acting antihypertensives, (%)	0 (0.0)	1 (0.5)	0 (0.0)	1 (0.2)	0.423
**Lipid lowering drugs, (%)**	192 (85.0)	160 (81.2)	78 (69.6)	430 (80.4)	0.004
Statins, (%)	187 (82.7)	154 (78.2)	75 (67.0)	416 (77.8)	0.004
Ezetimibe, (%)	31 (13.7)	11 (5.6)	15 (13.4)	57 (10.7)	0.015
Fibrates, (%)	26 (11.5)	16 (8.1)	7 (6.3)	49 (9.2)	0.236
Omega-3 fatty acids, (%)	1 (0.4)	0 (0.0)	2 (1.8)	3 (0.6)	0.124
PCSK9i, (%)	0 (0.0)	0 (0.0)	0 (0.0)	0 (0.0)	--

ACEIs: angiotensin-converting enzyme inhibitors; ARBs: angiotensin receptor blockers; dapa: dapagliflozin; PCSK9i: proprotein convertase subtilisin/kexin type 9 inhibitors; SOC: standard of care.

Supplementary Table S4. Changes in concomitant treatments between groups during the study period.

Variation between V1 and V0		SOC + inertia arms	Dapa arm	P
Variation of other antidiabetic drugs	Initiation, n (%)	32 (10.4)	20 (8.8)	0.46
	Withdrawal, n (%)	1 (0.3)	0 (0.0)	
	Never took the drug, n (%)	274 (88.7)	206 (91.2)	
	Continue taking the drug, n (%)	2 (0.6)	0 (0.0)	
Variation of pioglitazone	Initiation, n (%)	3 (1.0)	0 (0.0)	0.137
	Withdrawal, n (%)	0 (0.0)	0 (0.0)	
	Never took the drug, n (%)	306 (99.0)	226 (100.0)	
	Continue taking the drug, n (%)	0 (0.0)	0 (0.0)	
Variation of alpha-glucosidase inhibitors	Initiation, n (%)	0 (0.0)	0 (0.0)	0.399
	Withdrawal, n (%)	14 (4.5)	7 (3.1)	
	Never took the drug, n (%)	295 (95.5)	219 (96.9)	
	Continue taking the drug, n (%)	0 (0.0)	0 (0.0)	
Variation of other antidiabetic drugs	Initiation, n (%)	0 (0.0)	0 (0.0)	--
	Withdrawal, n (%)	0 (0.0)	0 (0.0)	
	Never took the drug, n (%)	0 (0.0)	0 (0.0)	
	Continue taking the drug, n (%)	0 (0.0)	0 (0.0)	
Variation of antihypertensive drugs	Initiation, n (%)	4 (1.3)	1 (0.4)	0.755
	Withdrawal, n (%)	5 (1.6)	3 (1.3)	
	Never took the drug, n (%)	95 (30.7)	73 (32.3)	
	Continue taking the drug, n (%)	205 (66.3)	149 (65.9)	
Variation of diuretics	Initiation, n (%)	11 (3.6)	3 (1.3)	0.289
	Withdrawal, n (%)	8 (2.6)	3 (1.3)	
	Never took the drug, n (%)	200 (64.7)	149 (65.9)	
	Continue taking the drug, n (%)	90 (29.1)	71 (31.4)	
Variation of ACEIs	Initiation, n (%)	13 (4.2)	5 (2.2)	0.4
	Withdrawal, n (%)	7 (2.3)	4 (1.8)	
	Never took the drug, n (%)	197 (63.8)	138 (61.1)	
	Continue taking the drug, n (%)	92 (29.8)	79 (35.0)	
Variation of ARBs	Initiation, n (%)	5 (1.6)	5 (2.2)	0.603
	Withdrawal, n (%)	6 (1.9)	5 (2.2)	
	Never took the drug, n (%)	213 (68.9)	165 (73.0)	
	Continue taking the drug, n (%)	85 (27.5)	51 (22.6)	
Variation of calcium channel blockers	Initiation, n (%)	8 (2.6)	0 (0.0)	0.11
	Withdrawal, n (%)	5 (1.6)	4 (1.8)	
	Never took the drug, n (%)	245 (79.3)	186 (82.3)	
	Continue taking the drug, n (%)	51 (16.5)	36 (15.9)	
Variation of beta blockers	Initiation, n (%)	5 (1.6)	2 (0.9)	0.579
	Withdrawal, n (%)	3 (1.0)	4 (1.8)	
	Never took the drug, n (%)	274 (88.7)	205 (90.7)	
	Continue taking the drug, n (%)	27 (8.7)	15 (6.6)	
Variation of alpha blockers	Initiation, n (%)	3 (1.0)	2 (0.9)	0.311
	Withdrawal, n (%)	2 (0.6)	3 (1.3)	
	Never took the drug, n (%)	297 (96.1)	220 (97.3)	
	Continue taking the drug, n (%)	7 (2.3)	1 (0.4)	
Variation of centrally acting antihypertensives	Initiation, n (%)	0 (0.0)	0 (0.0)	0.392
	Withdrawal, n (%)	0 (0.0)	0 (0.0)	
	Never took the drug, n (%)	308 (99.7)	226 (100.0)	
	Continue taking the drug, n (%)	1 (0.3)	0 (0.0)	
Variation of lipid lowering drugs	Initiation, n (%)	8 (2.6)	6 (2.7)	0.019
	Withdrawal, n (%)	2 (0.6)	5 (2.2)	

	Never took the drug, n (%)	69 (22.3)	29 (12.8)	
	Continue taking the drug, n (%)	230 (74.4)	186 (82.3)	
Variation of statins	Initiation, n (%)	10 (3.2)	8 (3.5)	0.035
	Withdrawal, n (%)	2 (0.6)	4 (1.8)	
	Never took the drug, n (%)	78 (25.2)	35 (15.5)	
	Continue taking the drug, n (%)	219 (70.9)	179 (79.2)	
Variation of ezetimibe	Initiation, n (%)	3 (1.0)	5 (2.2)	0.022
	Withdrawal, n (%)	4 (1.3)	10 (4.4)	
	Never took the drug, n (%)	279 (90.3)	185 (81.9)	
	Continue taking the drug, n (%)	23 (7.4)	26 (11.5)	
Variation of fibrates	Initiation, n (%)	2 (0.6)	3 (1.3)	0.136
	Withdrawal, n (%)	0 (0.0)	2 (0.9)	
	Never took the drug, n (%)	286 (92.6)	198 (87.6)	
	Continue taking the drug, n (%)	21 (6.8)	23 (10.2)	
Variation of omega-3 fatty acids	Initiation, n (%)	0 (0.0)	0 (0.0)	0.481
	Withdrawal, n (%)	0 (0.0)	1 (0.4)	
	Never took the drug, n (%)	307 (99.4)	224 (99.1)	
	Continue taking the drug, n (%)	2 (0.6)	1 (0.4)	
Variation of PCSK9i	Initiation, n (%)	0 (0.0)	0 (0.0)	--
	Withdrawal, n (%)	0 (0.0)	0 (0.0)	
	Never took the drug, n (%)	309 (100.0)	226 (100.0)	
	Continue taking the drug, n (%)	0 (0.0)	0 (0.0)	
**Variation between V1 and V2**		**SOC + inertia arms**	**Dapa arm**	**P**
Variation of other antidiabetic drugs	Initiation, n (%)	20 (27.0)	9 (22.5)	0.647
	Withdrawal, n (%)	1 (1.4)	0 (0.0)	
	Never took the drug, n (%)	53 (71.6)	31 (77.5)	
	Continue taking the drug, n (%)	0 (0.0)	0 (0.0)	
Variation of pioglitazone	Initiation, n (%)	3 (1.0)	0 (0.0)	0.137
	Withdrawal, n (%)	0 (0.0)	0 (0.0)	
	Never took the drug, n (%)	306 (99.0)	226 (100.0)	
	Continue taking the drug, n (%)	0 (0.0)	0 (0.0)	
Variation of alpha-glucosidase inhibitors	Initiation, n (%)	0 (0.0)	1 (0.4)	0.356
	Withdrawal, n (%)	14 (4.5)	7 (3.1)	
	Never took the drug, n (%)	295 (95.5)	218 (96.5)	
	Continue taking the drug, n (%)	0 (0.0)	0 (0.0)	
Variation of other antidiabetic drugs	Initiation, n (%)	0 (0.0)	0 (0.0)	--
	Withdrawal, n (%)	0 (0.0)	0 (0.0)	
	Never took the drug, n (%)	0 (0.0)	0 (0.0)	
	Continue taking the drug, n (%)	0 (0.0)	0 (0.0)	
Variation of antihypertensive drugs	Initiation, n (%)	1 (1.4)	5 (12.5)	0.031
	Withdrawal, n (%)	19 (25.7)	14 (35.0)	
	Never took the drug, n (%)	22 (29.7)	7 (17.5)	
	Continue taking the drug, n (%)	32 (43.2)	14 (35.0)	
Variation of diuretics	Initiation, n (%)	5 (1.6)	1 (0.4)	0.261
	Withdrawal, n (%)	86 (27.8)	70 (31.0)	
	Never took the drug, n (%)	206 (66.7)	151 (66.8)	
	Continue taking the drug, n (%)	12 (3.9)	4 (1.8)	
Variation of ACEIs	Initiation, n (%)	1 (0.3)	4 (1.8)	0.021
	Withdrawal, n (%)	86 (27.8)	80 (35.4)	
	Never took the drug, n (%)	209 (67.6)	139 (61.5)	
	Continue taking the drug, n (%)	13 (4.2)	3 (1.3)	
Variation of ARBs	Initiation, n (%)	1 (0.3)	2 (0.9)	0.342
	Withdrawal, n (%)	79 (25.6)	52 (23.0)	

	Never took the drug, n (%)<br>Continue taking the drug, n (%)	217 (70.2)<br>12 (3.9)	168 (74.3)<br>4 (1.8)	
Variation of calcium channel blockers	Initiation, n (%)<br>Withdrawal, n (%)<br>Never took the drug, n (%)<br>Continue taking the drug, n (%)	8 (2.6)<br>53 (17.2)<br>245 (79.3)<br>3 (1.0)	1 (0.4)<br>38 (16.8)<br>185 (81.9)<br>2 (0.9)	0.296
Variation of beta blockers	Initiation, n (%)<br>Withdrawal, n (%)<br>Never took the drug, n (%)<br>Continue taking the drug, n (%)	5 (1.6)<br>25 (8.1)<br>274 (88.7)<br>5 (1.6)	2 (0.9)<br>18 (8.0)<br>205 (90.7)<br>1 (0.4)	0.53
Variation of alpha blockers	Initiation, n (%)<br>Withdrawal, n (%)<br>Never took the drug, n (%)<br>Continue taking the drug, n (%)	0 (0.0)<br>8 (2.6)<br>300 (97.1)<br>1 (0.3)	1 (0.4)<br>2 (0.9)<br>221 (97.8)<br>2 (0.9)	0.247
Variation of centrally acting antihypertensives	Initiation, n (%)<br>Withdrawal, n (%)<br>Never took the drug, n (%)<br>Continue taking the drug, n (%)	0 (0.0)<br>0 (0.0)<br>308 (99.7)<br>1 (0.3)	0 (0.0)<br>0 (0.0)<br>226 (100.0)<br>0 (0.0)	0.392
Variation of lipid lowering drugs	Initiation, n (%)<br>Withdrawal, n (%)<br>Never took the drug, n (%)<br>Continue taking the drug, n (%)	9 (12.2)<br>23 (31.1)<br>14 (18.9)<br>28 (37.8)	3 (7.5)<br>13 (32.5)<br>5 (12.5)<br>19 (47.5)	0.619
Variation of statins	Initiation, n (%)<br>Withdrawal, n (%)<br>Never took the drug, n (%)<br>Continue taking the drug, n (%)	8 (2.6)<br>197 (63.8)<br>80 (25.9)<br>24 (7.8)	3 (1.3)<br>167 (73.9)<br>40 (17.7)<br>16 (7.1)	0.073
Variation of ezetimibe	Initiation, n (%)<br>Withdrawal, n (%)<br>Never took the drug, n (%)<br>Continue taking the drug, n (%)	8 (2.6)<br>24 (7.8)<br>274 (88.7)<br>3 (1.0)	7 (3.1)<br>33 (14.6)<br>183 (81.0)<br>3 (1.3)	0.075
Variation of fibrates	Initiation, n (%)<br>Withdrawal, n (%)<br>Never took the drug, n (%)<br>Continue taking the drug, n (%)	3 (1.0)<br>19 (6.1)<br>285 (92.2)<br>2 (0.6)	3 (1.3)<br>24 (10.6)<br>198 (87.6)<br>1 (0.4)	0.284
Variation of omega-3 fatty acids	Initiation, n (%)<br>Withdrawal, n (%)<br>Never took the drug, n (%)<br>Continue taking the drug, n (%)	0 (0.0)<br>2 (0.6)<br>307 (99.4)<br>0 (0.0)	0 (0.0)<br>2 (0.9)<br>224 (99.1)<br>0 (0.0)	0.753
Variation of PCSK9i	Initiation, n (%)<br>Withdrawal, n (%)<br>Never took the drug, n (%)<br>Continue taking the drug, n (%)	0 (0.0)<br>0 (0.0)<br>309 (100.0)<br>0 (0.0)	0 (0.0)<br>0 (0.0)<br>226 (100.0)<br>0 (0.0)	--

ACEis: angiotensin-converting enzyme inhibitors; ARBs: angiotensin receptor blockers; dapa: dapagliflozin; PCSK9i: proprotein convertase subtilisin/kexin type 9 inhibitors; SOC: standard of care.

Supplementary Table S5. Changes in anthropometric data between groups during the study period.

**Variation between V1 and V0**	**SOC + inertia arms**	**Dapa arm**	**Mean difference between groups**	**Cohen's d**	**P**
BMI, Kg/m^2	7.7 (18.0)	5.3 (12.5)	3.7	0.1	0.066
Body weight, Kg	0.2 (4.7)	-1.1 (5.2)	1.6	0.5	0.002
Waist circumference, cm	0.8 (5.2)	-1.5 (3.1)	2.7	0.9	<0.001
Waist-to-height ratio	0.5 (3.0)	-0.8 (2.8)	1.7	0.7	<0.001
Body fat percentage	1.3 (14.3)	-0.4 (1.6)	0.9	0.8	0.131
SBP, mmHg	1.7 (15.0)	-3.7 (12.7)	6.2	0.7	<0.001
DBP, mmHg	0.4 (9.8)	-2.0 (8.8)	3.4	0.5	0.004
Pulse pressure, mmHg	1.8 (12.1)	-1.2 (13.9)	3.2	0.4	0.009
Heart rate, bpm	0.1 (8.6)	-2.1 (7.3)	2.7	0.4	0.003
**Variation between V1 and V2**	**SOC + inertia arms**	**Dapa arm**	**Mean difference between groups**	**Cohen's d**	**P**
BMI, Kg/m^2	0.5 (5.5)	-0.1 (5.6)	0.8	0.1	0.250
Body weight, Kg	0.1 (7.6)	-3.2 (4.5)	3.8	0.6	<0.001
Waist circumference, cm	0.4 (4.2)	-3.6 (4.6)	4.4	1.0	<0.001
Waist-to-height ratio	0.2 (3.2)	-2.1 (3.0)	2.7	1.0	<0.001
Body fat percentage	0.6 (2.1)	-0.9 (1.7)	1.4	0.9	<0.001
SBP, mmHg	2.9 (14.6)	-6.5 (11.7)	11.4	0.9	<0.001
DBP, mmHg	1.2 (9.2)	-3.2 (7.9)	5.9	0.7	<0.001
Pulse pressure, mmHg	1.9 (13.3)	-2.9 (13.6)	5.5	0.4	<0.001
Heart rate, bpm	1.5 (9.5)	-2.3 (7.9)	4.0	0.5	<0.001

BMI: body mass index; Dapa: dapagliflozin; DBP: diastolic blood pressure; SBP: systolic blood pressure; SOC: standard of care.

Supplementary Table S6. Changes in biochemical parameters between groups during the study period.

Variation between V1 and V0	SOC + inertia arms	Dapa arm	Mean difference between groups	Cohen's d	P
FPG (mg/dL), mean (SD)	-5.3 (38.6)	-18.3 (48.3)	13.6	0.3	0.001
HbA1c (%), mean (SD)	-0.2 (0.9)	-0.6 (1.2)	0.5	0.5	<0.001
Total cholesterol (mg/dL), mean (SD)	-4.8 (37.0)	-7.0 (37.5)	2.3	0.1	0.504
HDL-c (mg/dL), mean (SD)	0.1 (8.2)	1.2 (8.1)	-0.6	0.1	0.161
LDL-c (mg/dL), mean (SD)	-4.4 (32.2)	-4.9 (32.7)	-0.3	0.01	0.878
TG (mg/dL), mean (SD)	-6.3 (66.4)	-16.3 (69.0)	11.0	0.2	0.095
Sodium (mEq/L), mean (SD)	0.2 (2.8)	0.1 (2.5)	0.1	0.02	0.831
Potassium (mEq/L), mean (SD)	0.0 (0.4)	0.01 (0.4)	0	0.03	0.724
SUA (mg/dL), mean (SD)	0.03 (0.9)	-0.3 (1.1)	0.3	0.3	0.005
AST (U/L), mean (SD)	0.5 (10.7)	-0.6 (12.3)	1.6	0.1	0.507
ALT (U/L), mean (SD)	-1.2 (13.4)	-3.0 (15.9)	2.7	0.1	0.183
GGT (U/L), mean (SD)	-0.02 (16.9)	-6.0 (22.7)	6.7	0.3	0.005
Creatinine (mg/dL), mean (SD)	0.01 (0.1)	0.01 (0.1)	0	0.1	0.486
eGFR (mL/min/1.73 m2), mean (SD)	-0.2 (14.2)	-2.2 (12.2)	1.1	0.2	0.088
uACR (mg/g), mean (SD)	0.9 (10.8)	-4.8 (23.9)	5.1	0.3	0.006
**Variation between V1 and V2**	**SOC + inertia arms**	**Dapa arm**	**Mean difference between groups**	**Cohen's d**	**P**
FPG (mg/dL), mean (SD)	-2.2 (43.9)	-26.5 (48.2)	24.4	0.5	<0.001
HbA1c (%), mean (SD)	0.1 (1.1)	-0.8 (1.2)	0.9	0.8	<0.001
Total cholesterol (mg/dL), mean (SD)	-9.0 (36.2)	-13.4 (37.9)	3.7	0.1	0.180
HDL-c (mg/dL), mean (SD)	-0.8 (9.1)	2.6 (8.6)	-2.8	0.4	<0.001
LDL-c (mg/dL), mean (SD)	-6.7 (31.7)	-11.8 (33.8)	3.0	0.2	0.088
TG (mg/dL), mean (SD)	-4.2 (83.5)	-24.1 (70.8)	23.3	0.3	0.005
Sodium (mEq/L), mean (SD)	0.2 (2.6)	0.2 (2.6)	0.1	0.02	0.857
Potassium (mEq/L), mean (SD)	0.0 (0.4)	-0.01 (0.4)	0.04	0.02	0.816
SUA (mg/dL), mean (SD)	0.04 (1.0)	-0.5 (1.1)	0.6	0.6	<0.001
AST (U/L), mean (SD)	-0.4 (10.5)	-2.7 (10.9)	2.8	0.2	0.145
ALT (U/L), mean (SD)	-1.8 (12.6)	-5.8 (15.2)	5.6	0.3	0.003
GGT (U/L), mean (SD)	0.9 (19.9)	-7.1 (26.4)	7.6	0.4	0.001
Creatinine (mg/dL), mean (SD)	0.03 (0.1)	0.0 (0.1)	0.03	0.3	0.001
eGFR (mL/min/1.73 m2), mean (SD)	-7.3 (14.5)	-6.6 (13.4)	-0.5	0.04	0.611
uACR (mg/g), mean (SD)	6.0 (27.3)	-0.1 (20.7)	7.5	0.3	0.025

ALT: alanine aminotransferase; AST: aspartate aminotransferase; Dapa: dapagliflozin; DBP: diastolic blood pressure; eGFR: estimated glomerular filtration rate; FGP: fasting plasma glucose; GGT: gamma-glutamyl transferase; HbA1c: glycated hemoglobin A1c; HDL-c: high-density lipoprotein cholesterol; LDL-c: low-density lipoprotein cholesterol; SD: standard deviation; SOC: standard of care; SUA: serum uric acid; TC: total cholesterol; TG: triglycerides; uACR: urine albumin-creatinine ratio.

Supplementary Table S7. Prevalence and degree of control of the main biochemical parameters.

	Dapa arm (n=226)	SOC arm (n=197)	Inertia arm (n=112)	Total (n=535)	P
FGP 90-130 mg/dL, n (%)	109 (48.2)	103 (52.3)	52 (46.4)	264 (49.3)	0.556
HbA1c <6.5%, n (%)	51 (22.6)	82 (41.8)	33 (29.5)	166 (31.1)	<0.001
HbA1c <7.0%, n (%)	116 (51.3)	128 (65.3)	55 (49.1)	299 (56.0)	0.004
HbA1c <7.5%, n (%)	160 (70.8)	168 (85.7)	81 (72.3)	409 (76.6)	0.001
HbA1c <8.0%, n (%)	199 (88.1)	182 (92.9)	95 (84.8)	476 (89.1)	0.073
Total cholesterol <200 mg/dL	185 (81.9)	162 (82.7)	85 (77.3)	432 (81.2)	0.485
HDLc ≥40 mg/dL (men), 50 mg/dL (women)	134 (60.9)	130 (68.8)	63 (58.3)	327 (63.2)	0.127
TG <150 mg/dL	120 (53.3)	133 (68.2)	67 (60.9)	320 (60.4)	0.008
Fatty Liver Index					0.157
<30	9 (5.8)	10 (7.8)	9 (15.0)	28 (8.1)	
30-59	37 (23.7)	37 (28.9)	12 (20.0)	86 (25.0)	
≥60	110 (70.5)	81 (63.3)	39 (65.0)	230 (66.9)	

Dapa: dapagliflozin; FGP: fasting plasma glucose; FLI: fatty liver index; HbA1c: glycated hemoglobin A1c; HDL-c: high-density lipoprotein cholesterol; SOC: standard of care; TG: triglycerides.

Supplementary Table S8. Achievement of the goal of reaching HbA1c levels <8.0%, <7.5%, <7.0% and <6.5% between groups in the different study visits.

HbA1c <8.0% in V0		Adjusted for age, residence setting, education level, employment status	
		**OR (95% CI)**	**P**
**SOC + Inertia arms, n (%)**	277 (89.9)	1 Reference	0.539
**DAPA arm, n (%)**	199 (88.1)	0.8 (0.5-1.5)	
**HbA1c <7.5% in V0**		**Adjusted for age, residence setting, education level, employment status**	
		**OR (95% CI)**	**P**
**SOC + Inertia arms, n (%)**	249 (80.8)	1 Reference	0.008
**DAPA arm, n (%)**	160 (70.8)	0.6 (0.4-0.9)	
**HbA1c <7.0% in V0**		**Adjusted for age, residence setting, education level, employment status**	
		**OR (95% CI)**	**P**
**SOC + Inertia arms, n (%)**	183 (59.4)	1 Reference	0.057
**DAPA arm, n (%)**	116 (51.3)	0.7 (0.5-1.0)	
**HbA1c <6.5% in V0**		**Adjusted for age, residence setting, education level, employment status**	
		**OR (95% CI)**	**P**
**SOC + Inertia arms, n (%)**	115 (37.3)	1 Reference	<0.001
**DAPA arm, n (%)**	51 (22.6)	0.5 (0.3-0.7)	
**HbA1c <8.0% in V2**		**Adjusted for age, residence setting, education level, employment status**	
		**OR (95% CI)**	**P**
**SOC + Inertia arms, n (%)**	260 (86.7)	1 Reference	0.021
**DAPA arm, n (%)**	208 (92.4)	2.1 (1.1-3.8)	
**HbA1c <7.5% in V2**		**Adjusted for age, residence setting, education level, employment status**	
		**OR (95% CI)**	**P**
**SOC + Inertia arms, n (%)**	224 (74.7)	1 Reference	0.270
**DAPA arm, n (%)**	176 (78.2)	1.3 (0.8-1.9)	
**HbA1c <7.0% in V2**		**Adjusted for age, residence setting, education level, employment status**	
		**OR (95% CI)**	**P**
**SOC + Inertia arms, n (%)**	168 (56.0)	1 Reference	0.748
**DAPA arm, n (%)**	132 (58.7)	1.1 (0.7-1.5)	
**HbA1c <6.5% in V2**		**Adjusted for age, residence setting, education level, employment status**	
		**OR (95% CI)**	**P**
**SOC + Inertia arms, n (%)**	107 (35.7)	1 Reference	0.209
**DAPA arm, n (%)**	69 (30.7)	0.8 (0.5-1.1)	

Dapa: dapagliflozin; HbA1c: glycated hemoglobin A1c; SOC: standard of care; OR: Odds Ratio; SBP: 95% CI: 95% confidence interval.

Supplementary Table S9. Achievement of the different cardiometabolic control objectives (HbA1c, body weight, SBP and/or FPG) between groups during the study period.

**Reduction HbA1c ≥0.5% + reduction SBP ≥2 mmHg between V1 and V0**		**Adjusted for age, residence setting, education level, employment status**	
		**OR (95% CI)**	**P**
**SOC + Inertia arms, n (%)**	21 (6.8)	1 Reference	<0.001
**DAPA arm, n (%)**	83 (36.9)	7.9 (4.6-13.5)	
**Reduction HbA1c ≥0.5% + reduction SBP ≥2 mmHg between V1 and V2**		**Adjusted for age, residence setting, education level, employment status**	
		**OR (95% CI)**	**P**
**SOC + Inertia arms, n (%)**	24 (7.9)	1 Reference	<0.001
**DAPA arm, n (%)**	110 (49.8)	12.4 (7.4-20.8)	
**Reduction HbA1c ≥0.5% + reduction body weight ≥2 kg between V1 and V0**		**Adjusted for age, residence setting, education level, employment status**	
		**OR (95% CI)**	**P**
**SOC + Inertia arms, n (%)**	16 (5.2)	1 Reference	<0.001
**DAPA arm, n (%)**	58 (25.7)	5.9 (3.2-10.7)	
**Reduction HbA1c ≥0.5% + reduction body weight ≥2 kg between V1 and V2**		**Adjusted for age, residence setting, education level, employment status**	
		**OR (95% CI)**	**P**
**SOC + Inertia arms, n (%)**	23 (7.6)	1 Reference	<0.001
**DAPA arm, n (%)**	103 (46.2)	10.4 (6.2-17.3)	
**Reduction SBP ≥2 mmHg + reduction body weight ≥2 kg between V1 and V0**		**Adjusted for age, residence setting, education level, employment status**	
		**OR (95% CI)**	**P**
**SOC + Inertia arms, n (%)**	22 (7.2)	1 Reference	<0.001
**DAPA arm, n (%)**	69 (30.8)	5.1 (3.0-8.7)	
**Reduction SBP ≥2 mmHg + reduction body weight ≥2 kg between V1 and V2**		**Adjusted for age, residence setting, education level, employment status**	
		**OR (95% CI)**	**P**
**SOC + Inertia arms, n (%)**	38 (12.7)	1 Reference	<0.001
**DAPA arm, n (%)**	101 (46.1)	5.7 (3.6-8.9)	
**FPG 90-130 mg/dL between V1 and V0**		**Adjusted for age, residence setting, education level, employment status**	
		**OR (95% CI)**	**P**
**SOC + Inertia arms, n (%)**	155 (50.2)	1 Reference	0.561
**DAPA arm, n (%)**	109 (48.2)	0.9 (0.6-1.3)	
**FPG 90-130 mg/dL between V1 and V2**		**Adjusted for age, residence setting, education level, employment status**	
		**OR (95% CI)**	**P**
**SOC + Inertia arms, n (%)**	143 (47.2)	1 Reference	0.959
**DAPA arm, n (%)**	109 (48.4)	1.0 (0.7-1.4)	

Dapa: dapagliflozin; FPG: fasting plasma glucose; HbA1c: glycated hemoglobin A1c; SOC: standard of care; OR: Odds Ratio; SBP: systolic blood pressure: 95% CI: 95% confidence interval.

Supplementary Table S10. Incidence of adverse events between V1 and V2.

	SOC + inertia arm	Dapa arm	Adjusted for age, residence setting, education level, employment status	
	n (%)	n (%)	OR (95% CI)	P
Incidence of headache	25 (8.1)	8 (3.5)	0.4 (0.2-1.0)	0.054
Incidence of cough	15 (4.9)	6 (2.7)	0.6 (0.2-1.7)	0.376
Incidence of nasopharyngitis	15 (4.9)	5 (2.2)	0.5 (0.2-1.5)	0.215
Incidence of genital infections	5 (1.6)	13 (5.8)	4.8 (1.5-15.3)	0.008
Incidence of urinary tract infections	21 (6.8)	13 (5.8)	1.0 (0.5-2.1)	0.994
Incidence of gastrointestinal adverse events	24 (7.8)	9 (4.0)	0.5 (0.2-1.2)	0.121
Incidence of ketoacidosis	0 (0.0)	0 (0.0)	NC	
Incidence of mild hypoglycemia	3 (1.0)	0 (0.0)	NC	
Incidence of severe hypoglycemia	0 (0.0)	0 (0.0)	NC	
Incidence of hypertransaminasemia	11 (3.6)	5 (2.2)	0.5 (0.2-1.5)	0.225
Incidence of fractures	3 (1.0)	3 (1.3)	0.8 (0.1-4.7)	0.833
Incidence of amputation of the lower extremities	0 (0.0)	0 (0.0)	NC	

NC: not computable because there were no cases in any of the groups. Incident event or comorbidity: new cases that did not exist in V1 and have appeared in the V1-V2 period. Dapa: dapagliflozin; OR: Odds Ratio; SOC: standard of care; 95% CI: 95% confidence interval.
